# Double reduction in allotetraploid peanut and the role of chromosomal imbalance in unexpected linkage map artifacts

**DOI:** 10.1093/g3journal/jkag269

**Published:** 2026-09-28

**Authors:** Samuele Lamon, Peter M Bourke, Brian L Abernathy, João F dos Santos, Ignácio J de Godoy, Soraya C M Leal-Bertioli, David J Bertioli

**Affiliations:** Institute of Plant Breeding, Genetics and Genomics, University of Georgia, Athens, GA 30602, United States; Plant Breeding, Wageningen University & Research, Wageningen PB 6708, The Netherlands; Center for Applied Genetic Technologies, University of Georgia, Athens, GA 30602, United States; Campinas Agronomical Institute, Botafogo, Campinas, SP 13075-630, Brazil; Campinas Agronomical Institute, Botafogo, Campinas, SP 13075-630, Brazil; Institute of Plant Breeding, Genetics and Genomics, University of Georgia, Athens, GA 30602, United States; Center for Applied Genetic Technologies, University of Georgia, Athens, GA 30602, United States; Department of Plant Pathology, University of Georgia, Athens, GA 30602, United States; Institute of Plant Breeding, Genetics and Genomics, University of Georgia, Athens, GA 30602, United States; Center for Applied Genetic Technologies, University of Georgia, Athens, GA 30602, United States; Department of Crop & Soil Sciences, University of Georgia, Athens, GA 30602, United States

**Keywords:** *Arachis hypogaea*, polyploidy, double reduction, homoeologous exchange, genetic instability, linkage mapping, segmental allopolyploidy

## Abstract

Polyploidization in peanut (*Arachis hypogaea* L.) fixed heterosis and also caused a genetic bottleneck isolating cultivated peanut from its wild diploid relatives. Mechanisms such as homoeologous exchange can partially restore genetic diversity by generating new allelic combinations. Double reduction is a rare polyploid-specific segregation pattern in which a single-dosage locus yields duplex gametes. It requires multivalent formation—associated with homoeologous exchange in allopolyploids—and crossing over between nonsister chromatids. Although peanut mainly exhibits disomic pairing, occasional multivalents theoretically allow low-frequency double reduction. To estimate double reduction and examine its relationship with genetic instability, we constructed a high-density phased linkage map (9,717 markers, 0.22 cM average spacing) from a backcross population between cultivated peanut and the neoallotetraploid [*A. magna* × *A. stenosperma*]^4×^ (MagSten). Initial maps showed distinctive double linkage group artifacts, and some progenies displayed abnormal genotyping patterns linked to unbalanced chromosomal compositions, outcomes of homoeologous exchange. Removing these progenies resolved the artifacts, revealing a previously unrecognized mapping artifact in allotetraploid peanut and suggesting a common origin for similar artifacts observed in other linkage maps. Analysis of the phased map detected double reduction in 12% of progenies; one event was confirmed as producing a genomic composition matching theoretical predictions, supporting the hypothesis that double reduction causes unbalanced genomic compositions in allopolyploids as well. These results indicate that double reduction is a rare but recurrent phenomenon in segmental allotetraploid peanut that, together with homoeologous exchange, contributes to genetic instability and generates new allelic combinations in this and likely other allopolyploid genomes.

## Introduction

Peanut (*Arachis hypogaea* L.) is an allotetraploid species (2*n* = 4× = 40; AABB-type genome) that originated less than 10,000 years ago through a spontaneous hybridization event involving two diploid wild *Arachis* species, *A. duranensis* Krapov. & W.C. Greg. (A-type genome) and *A. ipaënsis* Krapov. & W.C. Greg. (B-type genome). The hybridization of these two wild species was likely facilitated by humans, who introduced *A. ipaënsis* into the growing habitat of *A. duranensis* (Bertioli et al. 2016, 2019; Blas et al. 2025).

Either through unreduced gametes or through hybridization followed by whole-genome duplication (García et al. 2020), cultivated peanut became tetraploid and genetically isolated from its wild diploid *Arachis* relatives. This event led to a significant genetic bottleneck in the peanut population, drastically reducing genetic diversity within the gene pool (Kochert et al. 1996; Seijo et al. 2007; Bertioli et al. 2011, 2019; Moretzsohn et al. 2013).

Despite this narrow origin, cultivated peanut evolved substantial morphological diversity and became a globally recognized crop. This was likely aided by polyploidization, which increased the response to selection relative to its diploid progenitors by combining divergent genomes and generating new selectable variation through homoeologous exchange (Mason and Wendel 2020; Lamon et al. 2025a). Nowadays, peanuts are classified into 2 subspecies: *hypogaea* and *fastigiata*, and 6 botanical varieties—*hypogaea* and *hirsuta* (within the *hypogaea* subspecies), and *fastigiata*, *vulgaris*, *aequatoriana*, and *peruviana* (in the *fastigiata* subspecies). Additionally, peanut encompasses 4 major cultivated market types: Runner, Spanish, Valencia, and Virginia, and thousands of landraces and cultivars, each one with its own distinct characteristics (Bertioli et al. 2011, 2019). Intriguingly, spontaneous genomic instability continues in modern peanut, with unbalanced chromosomal compositions such as BBBB, ABBB, AAAB, and AAAA configurations observed at rates of 1% or more (Lamon et al. 2025b).

Double reduction (DR) is a mechanism that we also propose can generate such imbalances in the progenies carrying the affected locus (Fig. 1). DR is a meiotic process, classically described in autopolyploid organisms, in which an individual carrying a locus in a single dosage produces duplex gametes for that same locus (Darlington 1929). DR increases homozygosity, can reduce the frequency of deleterious alleles in a population over time through negative selection, and has the potential to increase rare advantageous alleles in plant material if combined with marker-assisted selection (Butruille and Boiteux 2000; Bourke et al. 2015). In order for DR to manifest, three main events need to occur: multivalent pairing of chromosomes during prophase I, followed by crossing over between nonsister chromatids, and a subsequent nondisjunctional separation whereby the two sister-chromatid copies of the focal allele comigrate into the same gamete (Mather 1936; Ronfort et al. 1998; De Silva et al. 2005; Huang et al. 2019). In allopolyploid species, such as peanut, multivalent formation requires homoeologous pairing and may involve homoeologous exchange.

**Fig. 1.**
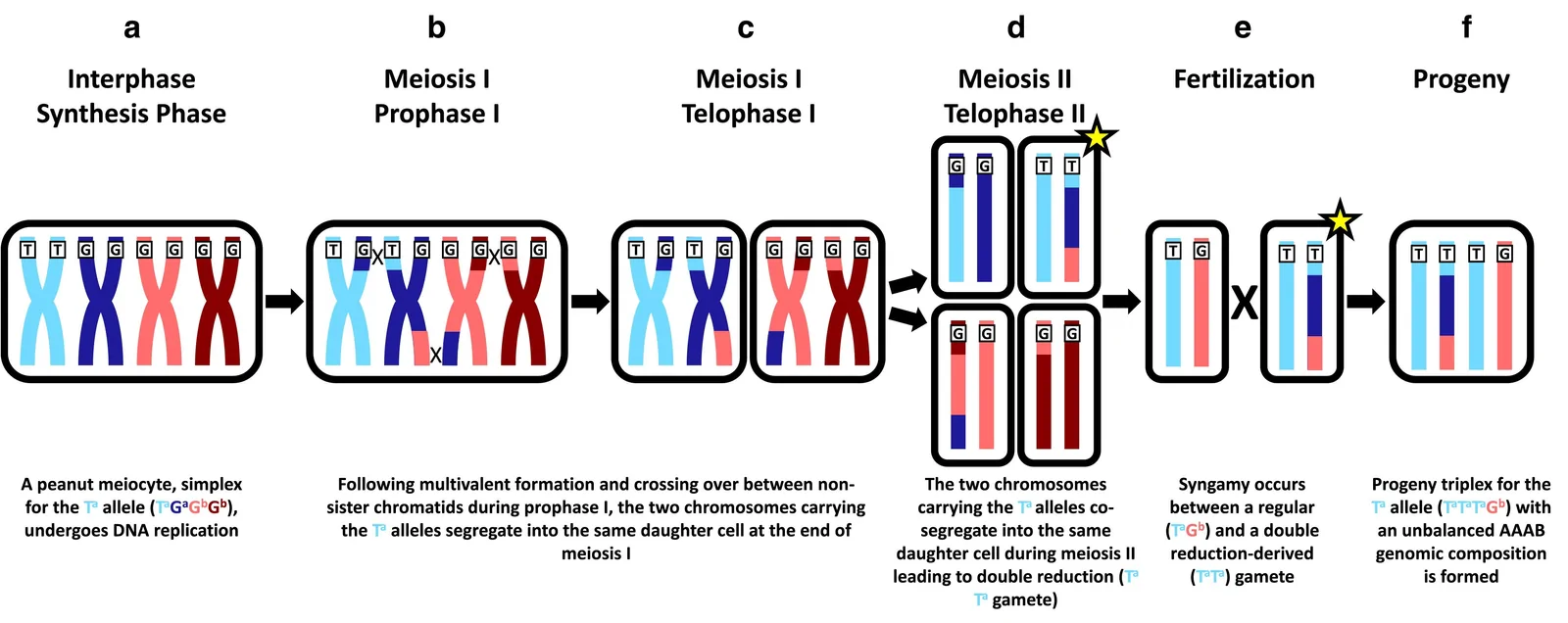
Cytological model of DR and the generation of unbalanced genomic compositions in segmental allotetraploid peanut. Letters “G” and “T” represent two different alleles. Blue and red colors, including their light and dark shades, as well as superscripts “a” and “b,” indicate the two peanut subgenomes, A and B, respectively. a) During the synthesis phase of interphase, a peanut meiocyte with a simplex genotype for the “T” allele (T^a^G^a^G^b^G^b^) undergoes DNA replication, forming sister chromatids. Although this heterozygous configuration is rare in peanut because of high subgenome homozygosity, the backcross population structure used in this study provides sufficient polymorphism for such configurations to occur (see Fig. 2). b) In prophase I, the segmental allopolyploid nature of peanut permits the occasional pairing of homologous and homoeologous chromosomes into a multivalent—a configuration that, in allopolyploids, is also the context in which homoeologous exchange can occur. Within this multivalent, crossing over takes place between nonsister chromatids. c) The multivalent configuration allows the two chromosomes carrying the “T” alleles to segregate together into the same daughter cell through nondisjunctional separation, which is not possible under strict disomic inheritance. d) During meiosis II, the two chromosomes carrying the “T” alleles segregate a second time into the same gamete, resulting in a gamete with two copies of the “T” allele, that is, a DR-derived gamete (highlighted by the yellow star). e) During fertilization, the DR-derived gamete is expected to fuse with a regular gamete from the other parent. Since peanut exhibits predominantly disomic inheritance, a T^a^T^a^G^b^G^b^ parent is expected to produce T^a^G^b^ gametes. f) In this scenario, the progeny has an unbalanced AAAB genomic composition (T^a^T^a^T^a^G^b^) at the locus under consideration.

Polysomic inheritance and multiple chromosomal pairing are typical of autopolyploid organisms since extremely high similarity is shared amongst different sets of chromosomes. In autopolyploids, ploidy increase takes place within a single species, often in a sole individual. Conversely, allopolyploids are formed by the hybridization of two different species and are also generally characterized by disomic inheritance (resulting from chromosome pairing within but not between subgenomes). In 2002, Ramsey and Schemske estimated that on average the percentages of multivalent occurrence in autopolyploid and allopolyploid plants are 28.8% and 8.0%, respectively. Even though multivalents are rarer in allopolyploids than autopolyploids, they arguably have a much higher biological significance in such species, given that recombination has a higher impact if genomes are more divergent (McGrath and Lynch 2012).

Detecting DR is a complex process that often requires a focus on specific markers. Similarly, accurately estimating DR rate can be challenging, frequently relying on computer simulations (Tai 1982; Haynes and Douches 1993; Bourke et al. 2015). Percentages of DR have been mainly detected and estimated in autopolyploid plants, eg purple loosestrife (*Lythrum salicaria* L.) (Fisher 1943; Fisher and Mather 1943), potato (*Solanum tuberosum* L.) (Haynes and Douches 1993; Bourke et al. 2015), great yellowcress (*Rorippa amphibia* [L.] Besser), yellow fieldcress (*R. sylvestris* [L.] Besser) (Stift et al. 2008), purple yam (*Dioscorea alata* L.) (Nemorin et al. 2012), clementine (*Citrus clementina* Hort. ex Tan.) (Aleza et al. 2016), hybrid tea rose (*Rosa hybrida* L.) (Bourke et al. 2017), chrysanthemum (*Chrysanthemum* × *morifolium* [Ramat.] Hemsl.) (Bourke et al. 2021), and much more rarely in animals such as rainbow trout (*Oncorhynchus mykiss* Walbaum) (Allendorf and Danzmann 1997) or Pacific oysters (*Crassostrea gigas* [Thunberg]) (Curole and Hedgecock 2005).

The detection and quantification of DR in allopolyploid organisms has been even more challenging. Cytological evidence consistent with DR has been reported in resynthesized *Brassica napus* (L.), where recombinant sister-chromatid fragments were observed segregating to the same pole during meiosis I, but the subsequent meiosis II segregation pattern and progeny-level inheritance of these products were not directly assessed (Xiong et al. 2021). Ahmed et al. (2020) estimated DR coefficients using a maximum-likelihood framework applied to the segregation of *Citrus micrantha*-diagnostic SNP markers in the allotetraploid “Giant Key” lime, a putative doubled diploid of “Mexican” lime carrying two homologs derived from *C. micrantha* and two from *C. medica*. To our knowledge, however, DR has not previously been resolved as discrete meiotic events in individual progeny of an established allopolyploid, nor has it been directly shown to generate genomic imbalance.

Peanut occupies an intermediate position along the auto- to allopolyploid spectrum, following a genetic model known as segmental allopolyploidy. As such, it exhibits mostly disomic pairing, with occasional multivalent formations and homoeologous exchanges (Husted 1933, 1936; Stalker 1981; Leal-Bertioli et al. 2015a; Lamon et al. 2025a, 2025b). These features make the occurrence of DR theoretically possible in peanut. However, due to the rarity of multivalents and high homozygosity in cultivated peanut, detecting and estimating DR remains challenging.

To overcome this limitation, we generated a BC_1_ population using the neoallotetraploid [*A. magna* K 30097 × *A. stenosperma* V 15076]^4×^ (MagSten) (Fávero et al. 2015) as the donor parent and “IAC OL4” (*A. hypogaea*) (Godoy et al. 2014) as the recurrent parent (Moretzsohn et al. 2023). “IAC OL4” is a cultivated Runner-type peanut variety developed for high yield, a short crop cycle, and other desirable traits, including high oleic acid content. MagSten was developed to transfer wild *Arachis* variation into cultivated peanut, including resistance to foliar fungal diseases such as late leaf spot and rust (Leal-Bertioli et al. 2015b; Moretzsohn et al. 2023).

Although MagSten is derived from diploid species belonging to the same broad A- and B-genome groups as the progenitors of cultivated peanut, these species are not the direct progenitors of *A. hypogaea*. The B-genome species used to generate MagSten, *A. magna*, is closely related to *A. ipaënsis*, whereas the A-genome species, *A. stenosperma*, belongs to the same genome group as *A. duranensis* but is distinct from it (Bertioli et al. 2016; Leal-Bertioli et al. 2024). This divergence provided sufficient polymorphism to track parental alleles and test for DR.

BC_1_ populations involving wild-derived neoallotetraploids are uncommon in peanut because the crop is predominantly self-pollinating and controlled crossing, although possible for research and breeding, is time-consuming. Population size can also be limited by fertility barriers and variable fertility in interspecific derivatives (Fávero et al. 2015; Moretzsohn et al. 2023). Thus, although the population used is limited in size, it was particularly useful for DR detection because it combined a cultivated allotetraploid background with segregating wild-derived segments.

Using this population, we detected and estimated DR and obtained direct evidence of a DR event associated with an AAAB genomic composition. Both theoretical considerations and our findings indicate that DR can result in unbalanced genomic compositions in the progenies (Fig. 1). To enable accurate estimation of DR, we constructed a high-density, phased genetic linkage map, which facilitated detailed analysis of allele inheritance patterns. Additionally, we identified double linkage group (LG) artifacts in Marey plots and showed that these artifacts resulted from large-scale unbalanced genomic compositions following homoeologous exchange, a scale of imbalance not attributable to DR. Resolving these artifacts improved the resolution of the genetic map and revealed that chromosomal imbalance caused by homoeologous exchange can produce characteristic LG artifacts in segmental allopolyploid species.

## Materials and methods

### Plant material

To enable analysis of introgression patterns and recombination between wild and cultivated genomes in a backcross framework, a BC_1_ population was developed using the neoallotetraploid [*A. magna* K 30097 × *A. stenosperma* V 15076]^4×^ (MagSten) (Fávero et al. 2015) as the donor parent and “IAC OL4” (*A. hypogaea*) (Godoy et al. 2014) as the recurrent parent (Moretzsohn et al. 2023). A total of 74 BC_1_ progenies, numbered from “Ind 1” to “Ind 74,” were included in the study. Additionally, BC_1_F_2_ progenies were derived by selfing from Ind 27, 40, and 41. These BC_1_F_2_ progenies were labeled based on single-seed descent identification. For example, Ind 40.2 indicates the second progeny of the BC_1_ plant Ind 40.

### Genotyping and sequencing

To obtain genome-wide marker data for linkage analysis and to assess genomic composition in selected progenies, DNA was extracted from young leaves of BC_1_ progenies, selected BC_1_F_2_ progenies, and “IAC OL4” and MagSten parentals. Genotyping was performed using the Axiom *Arachis* 48K SNP array v2 (Affymetrix, Santa Clara, CA, USA) (Pandey et al. 2017; Korani et al. 2019). In addition, four BC_1_ progenies (Ind 33, 39, 47, and 62) and two BC_1_F_2_ progenies (Ind 40.3 and 40.4) underwent whole-genome sequencing at 30× coverage using PCR-free Illumina NovaSeq PE150 technology (Novogene Co., Sacramento, CA, USA).

### Genotypic data scoring and linkage mapping

To construct a high-density linkage map and achieve consistent marker phasing between subgenomes, particular attention was given to genotype calling accuracy, segregation type interpretation, and marker filtering, especially in the absence of F_1_ genotype data. Therefore, raw cell intensity files from the Axiom *Arachis* 48K SNP array v2 were processed using the Axiom Analysis Suite (Thermo Fisher Scientific, Waltham, MA, USA) to generate a summarized signal intensity file, which was then imported into RStudio for further analysis (Posit Team 2025). SNP markers for tetraploid samples were scored on a tetraploid scale (0 to 4) using the “fitPoly” package (Voorrips et al. 2011, 2018), with “IAC OL4” and MagSten given as parents of the BC_1_ population, as the F_1_ hybrid genotypic data were unavailable (Supplementary Table 1).

Marker filtering and linkage mapping were performed using “polymapR” (Bourke et al. 2018). Initially, markers were filtered for nonmissing values in the parents, retaining 25,315 markers (Supplementary Fig. 1a). Subsequently, nonsegregating markers were removed and dosages were converted to basic segregating types, resulting in a final set of 11,565 markers (Supplementary Fig. 1b). Next, markers and individuals of the BC_1_ population were filtered for missing values, applying a cutoff rate of 0.25 for both. The allele dosage matrix was then filtered for duplicated individuals (cutoff value 0.95), identifying four pairs of duplicated individuals and four individuals resulting from self-pollination in “IAC OL4.” After merging these cases, 66 individuals remained for linkage mapping. Finally, the allele dosage matrix was filtered for duplicated markers, retaining 5,225 unique markers (Supplementary Fig. 1c).

Most markers were initially scored by “fitPoly” as simplex × nulliplex and nulliplex × simplex types (Supplementary Fig. 1, b and c). However, because cultivated peanut is characterized by extremely high subgenome homozygosity, simplex × nulliplex markers are expected to be very rare. As MagSten was entered in place of the F_1_ hybrid as the pollen parent for genotype calling, it is more accurate to interpret these simplex × nulliplex markers as duplex × simplex segregation types (Fig. 2a). For further mapping purposes, all markers erroneously called as simplex × nulliplex were relabeled as nulliplex × simplex.

**Fig. 2.**
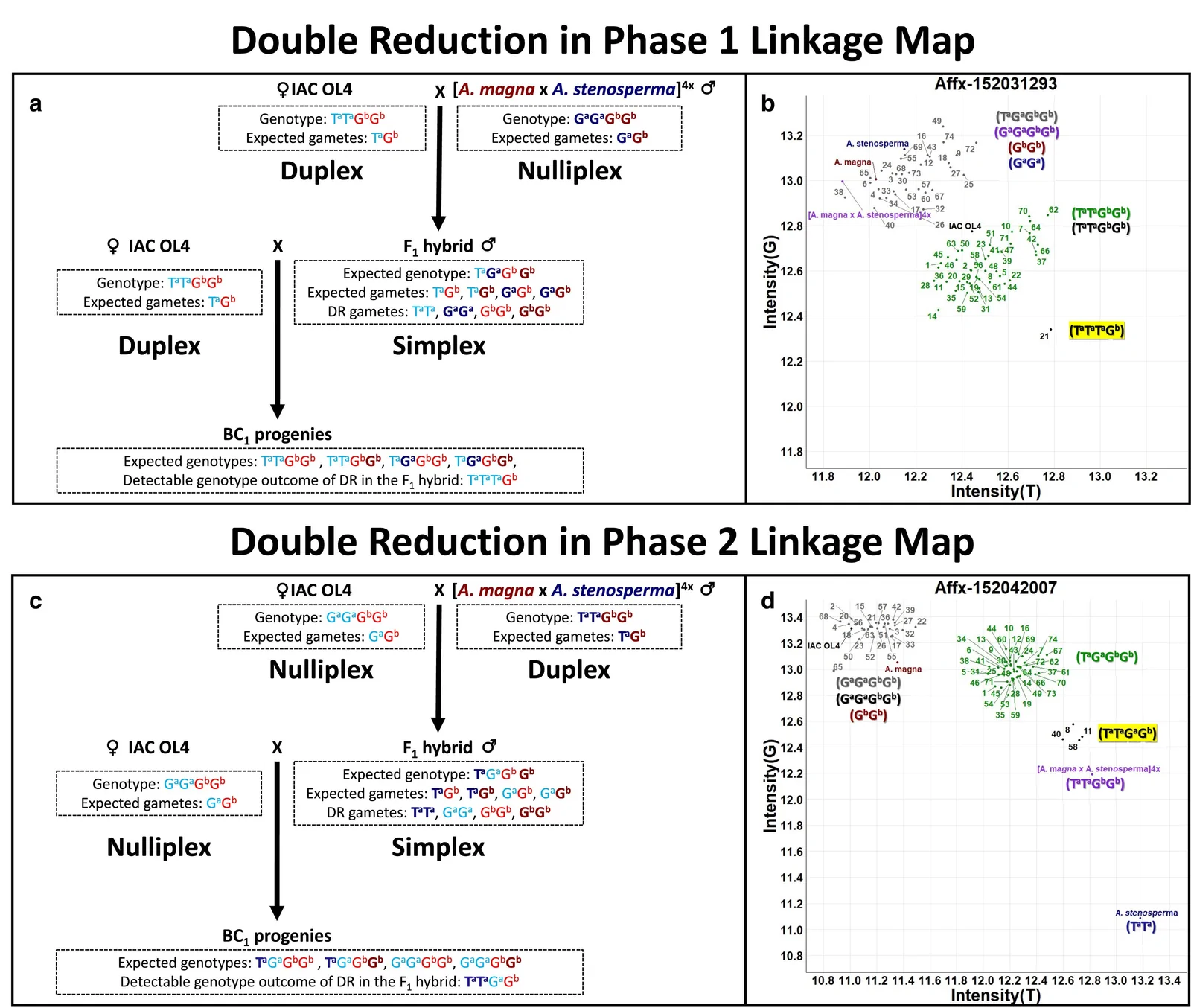
Detection of DR in the BC_1_ population. Diagrams show DR events in the “IAC OL4” × F_1_ hybrid BC_1_ population: (a) and (c) depict the marker logic for detecting DR in linkage map phases 1 and 2, respectively, while (b) and (d) show the corresponding SNP intensity data. “G” and “T” indicate alleles, with superscripts “a” and “b” denoting peanut subgenomes A and B. In (a) and (c), colors indicate *Arachis* genome species combinations: “IAC OL4” (light blue/red for A/B subgenome), *A. magna* (B; dark red), and *A. stenosperma* (A; dark blue); in (b) and (d), numbers label BC_1_ progenies, with the *y*-axis showing “G” allele intensity and the *x*-axis “T” allele intensity. In both phases, the F_1_ hybrid is simplex for “T” (T^a^G^a^G^b^G^b^), differing only in whether the T allele was inherited from “IAC OL4” (phase 1) or from MagSten (phase 2), with parental dosages reversed accordingly: in phase 1, “IAC OL4” is duplex (T^a^T^a^G^b^G^b^) and MagSten nulliplex (G^a^G^a^G^b^G^b^); in phase 2, this is reversed. Under disomic inheritance, the F_1_ produces T^a^G^b^ or G^a^G^b^ gametes in both phases, but because “IAC OL4” contributes a different gamete in each phase (T^a^G^b^ in phase 1, G^a^G^b^ in phase 2), the expected progeny scores differ: simplex or duplex (T^a^G^a^G^b^G^b^/T^a^T^a^G^b^G^b^) in phase 1 vs nulliplex or simplex (G^a^G^a^G^b^G^b^/T^a^G^a^G^b^G^b^) in phase 2. A DR-derived T^a^T^a^ gamete from the F_1_, however, produces an unbalanced AAAB progeny in both cases, falling outside either expected pair—scored as triplex (T^a^T^a^T^a^G^b^) in phase 1 or duplex (T^a^T^a^G^a^G^b^) in phase 2. SNP intensities for representative markers (Affx-152031293, phase 1; Affx-152042007, phase 2) are shown in (b) and (d), respectively, with DR-derived AAAB progenies highlighted in yellow in each.

Markers were aligned against A and B subgenomes of the “Tifrunner” (*A. hypogaea*) reference genome (version gnm2) to determine their physical positions (Bertioli et al. 2019). The alignment process was conducted in the Unix environment.

Pairwise recombination frequency, LOD scores, and linkage phases were calculated for markers using the “polymapR” linkage() function with ploidy equal to 2, with an expectation of 20 LGs. Marker clusters were generated over a range of LOD thresholds, with a minimum cluster size of 11 markers; at a threshold of 11, unique markers were clustered into 20 groups. Concordance of marker positions across the two reference assemblies, along with the application of the phase_SN_diploid() function, was used to phase markers between the A and B subgenomes. Inter-LG linkage signal was then assessed and duplicated markers were reintegrated, yielding a total of 9,717 markers (Supplementary Table 2).

The classification of markers during genotype calling into duplex × simplex (relabeled as nulliplex × simplex for mapping purposes) and nulliplex × simplex effectively phased markers between the two parental lines, with duplex × simplex corresponding to phase 1 (“IAC OL4”) and nulliplex × simplex corresponding to phase 2 (F_1_ hybrid). A very high concordance was observed between “genotype calling phasing” and linkage map phasing (Supplementary Fig. 2).

Summary statistics were calculated for the integrated map, and Marey plots were generated to illustrate the relationship between genetic distances and the physical positions on the “Tifrunner” reference genome, with genetic positions reoriented where necessary to match the physical map; pericentromeric regions were highlighted. Out of 9,717 markers, 1,628 were excluded from the plots because their corresponding subgenomes lacked “Tifrunner” physical positions. The plots revealed unexpected double-LG artifacts in LGs 4 and 14, with the artifact in LG14 being so pronounced that it resulted in the formation of two distinct LGs.

### Interspersed genotyping patterns and Marey plots resolution

During linkage map construction, we observed pronounced mapping artifacts in some LGs, including the appearance of doubled lines and even an entire doubled LG in Marey plots. These artifacts suggested that a subset of progenies might violate common assumptions (such as balanced tetraploid inheritance) used for linkage analysis. To investigate this, we systematically analyzed phased genotypic data to identify regions exhibiting interspersed allele dosage patterns indicative of unbalanced genomic compositions.

After initial map refinement by removing markers with missing alleles or lacking physical alignment information, the integrated map was divided into two phases. Examination of the phased maps—particularly phase 2—revealed extensive interspersed genotyping patterns, characterized by rapid switches in inferred allele dosage between consecutive markers ordered by physical position. Such interspersed genotyping patterns are indicative of unbalanced polyploid states (eg AAAB or ABBB) that produce allele dosage states incompatible with the genotyping model assumptions and have previously been described by Lamon et al. (2025a). In that study, unbalanced genotypic compositions were resolved by scoring genotypes on a tetraploid scale using “fitPoly” and restricting analyses to markers with clear A- and B-subgenome differentiation. In the present study, however, resolving interspersed genotyping proved more challenging.

Although BC_1_ progenies were scored on a tetraploid scale, the mapping population comprised four distinct subgenomic sources: A and B subgenomes from the cultivated parent (“IAC OL4”) and A and B subgenomes from the wild parents (*A. stenosperma* and *A. magna*). In phase 1, A- and B-subgenome alleles were distinguishable in the cultivated parent but identical in the wild parent, whereas the opposite configuration occurred in phase 2 (Fig. 2). This phase-dependent subgenomic symmetry reduced effective dosage resolution and likely contributed to interspersed genotyping patterns, similar to those previously reported, complicating the identification of progenies with unbalanced genomic compositions.

To systematically identify such cases, we developed an algorithm to detect regions of interspersed genotyping. Phased colormaps were first converted into binary matrices indicating whether allele dosage changed between consecutive markers. A sliding window of 11 markers (5 upstream, the focal marker, and 5 downstream) was applied; if more than 4 dosage changes occurred within the window, the central marker was classified as part of an interspersed genotyping region. These regions were visualized as chromosome-level heatmaps, and the number of interspersed markers per chromosome was summarized using bar plots.

Interspersed genotyping was most pronounced on LG14 of phase 2, which was also the LG exhibiting strong double-LG artifacts in Marey plots. To test whether these artifacts were caused by progenies with abnormal genomic composition, we quantified interspersed genotyping on LG14 (phase 2) for each BC_1_ progeny. This analysis identified 18 progenies with markedly elevated interspersed patterns (Supplementary Fig. 3a).

Linkage mapping was then repeated after excluding these progenies. This intervention resulted in the complete disappearance of the double-LG artifacts, particularly on LGs 4 and 14, and restored coherent Marey plot structure. These interspersed genotyping patterns were later shown to be due to large-scale unbalanced genomic compositions.

### Estimation of DR

To identify genomic regions showing signatures of DR and distinguish these from alternative recombination scenarios, the integrated linkage map was refined by removing markers with missing alleles or lacking physical alignment information and subsequently divided into the two phases. Regions showing DR-type segregation—in which an allele present in single copy in only one parent appeared in double copy in a progeny—were identified. While similar patterns could also result from tetrasomic recombination in the female parent during the backcross in phase 1 or from 2 consecutive rounds of tetrasomic recombination in the male parent in phase 2, DR provides the most parsimonious explanation for the observed patterns, especially in phase 2 (Fig. 2).

To minimize the impact of genotyping errors, only regions where multiple consecutive markers displayed DR scores were considered as true DR events. This filtering step was performed manually through visual inspection of phased colormaps. Final DR inferences for both phases were visualized as heatmaps using “ggplot2” package (Wickham 2016), providing a chromosome-level overview of DR event distribution.

### DR estimation using a hidden Markov model

To independently validate DR inference using a probabilistic framework that allows for multivalent pairing, we explored an alternative approach based on estimating posterior genotype probabilities using a hidden Markov model (Zheng et al. 2016). However, such an approach would require an integrated genetic linkage map, which is not possible to create due to lack of linkage between subgenomes (eg LG1 and LG11 are syntenic but cannot be combined into a single linkage map). To circumvent this difficulty, we used a physical-assembly guided approach to create a pseudo-integrated linkage map of 10 groups via centromere alignment. First, pericentromeric regions were identified visually from Marey plots (genetic vs physical marker positions) using consensus physical positions (Supplementary Fig. 4a). The median genetic position of markers within these centromeres was taken as the approximate genetic position of the centromeres, and for each chromosome, one of the maps was shifted upwards so that centromeres were genetically aligned (Supplementary Fig. 4b). A pseudo-integrated map was created by merging both maps and resorting markers on the new consensus genetic positions (Supplementary Figs. 4c and 5). This was converted to a phased map by concatenating the phase information per chromosome across subgenomes into a “pseudo-autotetraploid” phased map (adding a dummy phase for parent 1, for which no segregating markers were present).

The pseudo-integrated phased map and marker genotypes were used to estimate posterior genotype identity by descent (IBD) probabilities in “polyqtlR” v.0.1.1 (Bourke et al. 2021) using the estimate_IBD() function with settings ploidy = 4 and bivalent_decoding = FALSE (ie allowing for the possibility of multivalent formation). These probabilities were subsequently re-estimated using the maxL_IBD() function using a range of genotyping error priors (*ɛ* = 0.01, 0.05, 0.1, 0.15, 0.2, 0.25, 0.3, 0.35). Maximum-likelihood estimates for the genotyping error rates per individual per chromosome were visualized in a heatmap.

### Sequencing data analysis

To assess subgenome balance and evaluate genomic composition in selected progenies, we first identified a robust set of subgenome-diagnostic polymorphisms. This was done using independent sources of short sequences aligned to a common reference genome: in silico-fragmented assemblies of *A. duranensis* (A genome) and *A. ipaënsis* (B genome), and Illumina whole-genome sequencing data from two independent sources. Using multiple sources ensured that subgenome-specific alleles were consistently identifiable and reliably recoverable from Illumina sequencing data in the progenies, following Lamon et al. (2025a). Illumina reads were quality-filtered using BBMap (v39.01) (Bushnell 2014) and, together with the in silico-fragmented assemblies, were aligned to the *A. ipaënsis* gnm1 reference genome using Burrows–Wheeler aligner (BWA) (v0.7.17-r1188) (Li and Durbin 2009), which provided a common coordinate framework. Variant calling was performed using BCFtools (version 1.15.1) (Danecek et al. 2021) to identify positions that consistently differentiated the A and B subgenomes. AB-diagnostic variant positions were retained only if they met stringent criteria, including concordance between sequencing datasets, a minimum of 95% consensus among mapped reads, sufficient read depth, and clear allele differentiation between the A and B subgenomes. This procedure yielded a fixed set of high-confidence subgenome-specific polymorphisms.

Genomic composition in progenies was inferred by calculating normalized counts of subgenome-diagnostic alleles at the predefined AB-diagnostic positions. Illumina whole-genome sequencing reads from selected BC_1_ and BC_1_F_2_ progenies (Ind 33, 39, 47, 62, 40.3, and 40.4) were then aligned to the same reference genome, and allele counts were extracted exclusively at these positions. This targeted interrogation allowed quantitative assessment of subgenome allele representation without de novo variant discovery. To normalize allele counts and account for potential mapping and coverage biases, synthetic tetraploid controls were generated by combining Illumina whole-genome sequencing reads from *A. duranensis* and *A. ipaënsis* in a 1:1 ratio (taking into account the different genome sizes). These synthetic datasets were processed identically to the progeny samples, and allele counts were calculated. Raw allele counts in the progenies were subsequently normalized using the formulas described in Lamon et al. (2025a), enabling direct comparison across samples. Normalized allele distributions for the selected BC_1_ and BC_1_F_2_ progenies were visualized using density plots generated with the “ggplot2” package in RStudio.

## Results

### Development of a high-density phased linkage map in peanut

A high-density phased linkage map was developed, distinguishing two phases in the BC_1_ population (Fig. 3). The phase 1 map included 3,194 SNP markers, while the phase 2 map contained 6,523 SNP markers, for a total of 9,717 mapped markers (Table 1 and Supplementary Table 2). On average, phase 1 had 160 markers per chromosome, with the highest density observed on LG2 (234 markers). Phase 2 averaged 326 markers per chromosome, peaking at 559 markers on LG2. The unequal marker density between phases should be considered when interpreting downstream DR detection. Because DR events were inferred manually from phased colormaps as genomic regions containing consecutive markers with DR-type segregation, the denser phase 2 map likely provided greater resolution for identifying such regions than the phase 1 map.

**Fig. 3.**
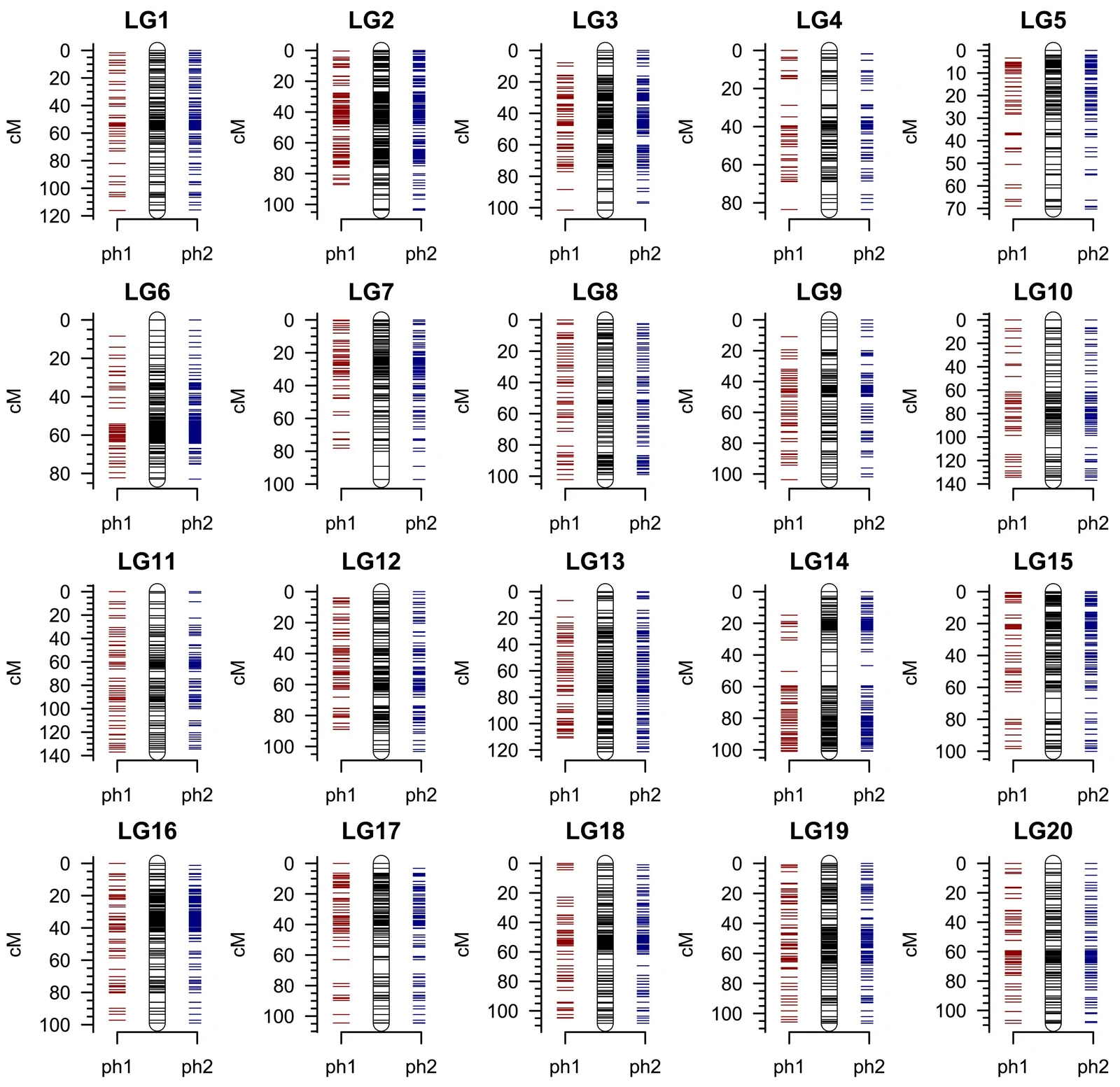
High-density phased linkage map of the BC_1_ population. Integrated linkage map (center, black) displaying all 9,717 phased SNP markers across 20 linkage groups (LGs 1 to 10, A subgenome; LGs 11 to 20, B subgenome), flanked by the two phase-specific maps: duplex × simplex markers (phase 1, left, red) and nulliplex × simplex markers (phase 2, right, blue).

**Table 1.** Summary statistics of the integrated linkage maps, including the number of SNP markers for phase 1 and phase 2, LG lengths, average marker distances, and maximum marker gap distances.

Subgenome	LG	No. of SNPs phase 1	No. of SNPs phase 2	LG length (cM)	Average distance (cM)	Maximum gap distance (cM)
A-subgenome	LG1	169	505	116.11	0.17	5.65
	LG2	234	559	103.74	0.13	6.25
	LG3	174	345	101.53	0.2	6.6
	LG4	118	145	83.54	0.32	7.55
	LG5	114	214	70.24	0.21	5.08
	LG6	146	337	82.93	0.17	5.54
	LG7	182	343	97.22	0.19	9.2
	LG8	166	305	102.24	0.22	4.13
	LG9	189	304	103.71	0.21	8.52
	LG10	139	295	136.88	0.32	10.43
B-subgenome	LG11	123	349	137.01	0.29	7.44
	LG12	127	244	103.21	0.28	5.56
	LG13	184	345	121.37	0.23	7.54
	LG14	169	277	100.94	0.23	9.04
	LG15	130	304	100.09	0.23	9.12
	LG16	157	298	99.00	0.22	5.77
	LG17	146	326	104.63	0.22	9.39
	LG18	207	361	108.44	0.19	4.41
	LG19	155	326	106.5	0.22	4.12
	LG20	165	341	108.75	0.21	4.78
Across the whole map		3,194	6,523	2,088.08	0.22	10.43

The integrated map covered a total genetic distance of 2,088.08 cM, with individual LG lengths ranging from 70.24 cM (LG5) to 137.01 cM (LG11). The average marker interval was 0.22 cM, while maximum distances between markers varied from 4.12 cM (LG19) to 10.43 cM (LG10). Strong cross-LG signals were found between the homoeologous groups LG3 and LG13, LG4 and LG14, and LG9 and LG19 that all have high collinearity (Supplementary Fig. 6).

Marey plots displayed distinct S-shapes with smooth transitions (Fig. 4a), indicative of a high-quality linkage map and consistent with expected recombination rate variation along the chromosomes. An exception was observed for LG5, which showed near-complete suppression of recombination along its upper arm. This pattern is consistent with the presence of a large post-polyploidy inversion in this region of *A. hypogaea* (Bertioli et al. 2019), one of the few large structural changes apparently characteristic of the cultivated species. This inversion likely stabilized the homoeologous chromosome pair by disrupting collinearity between the A and B subgenomes and would also be expected to suppress recombination between cultivated and wild chromosome 05 homologs. In addition, LG4 and LG14 showed extreme “double-line” artifacts. LG14 was particularly affected, presenting as two complete S-shaped curves.

**Fig. 4.**
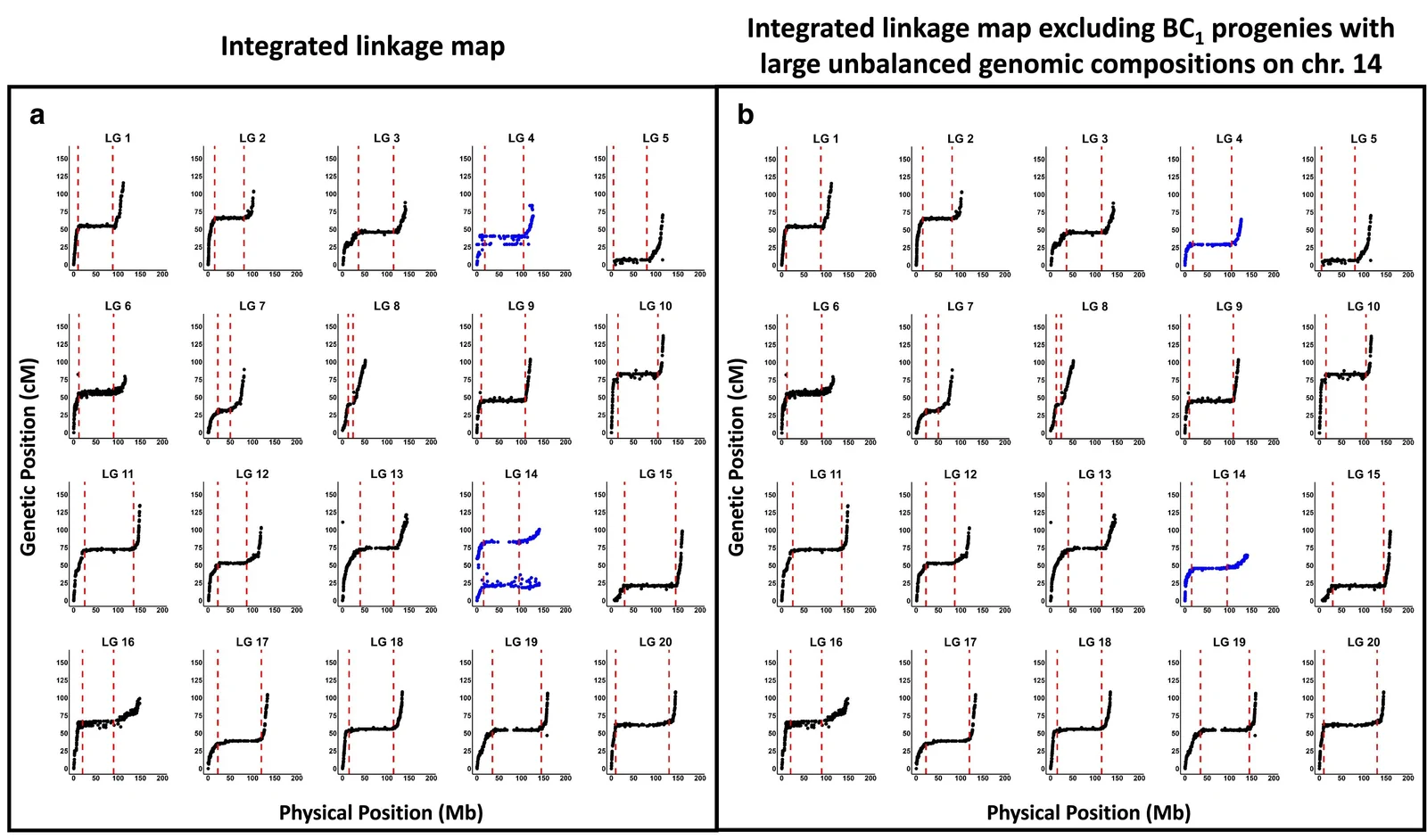
Marey plots before and after removal of progenies with unbalanced genomic compositions. Marey plots of the integrated linkage map (a) and of the map after excluding the 18 affected BC_1_ progenies on LG 14 (b). The *y*-axis represents genetic positions, and the *x*-axis corresponds to physical positions in the “Tifrunner” reference genome. In (a), double-LG artifacts are evident, particularly on LGs 4 and 14 (blue). These artifacts disappear in (b) following the exclusion of the affected progenies. Pericentromeric regions are indicated by red dashed lines.

### Genotyping and sequencing analyses reveal a correlation between interspersed genotyping patterns and unbalanced genomic compositions, which significantly affect linkage mapping

In this section, we describe how we discovered that two initially unexpected observations—chromosome-scale interspersed SNP genotyping patterns and the appearance of “doubled” LGs—were related, and we identify their underlying cause.

Regions with interspersed genotyping patterns were most frequently observed in chromosome set 4 (LGs 4 and 14), followed by sets 2 (LGs 2 and 12), 6 (LGs 6 and 16), 3 (LGs 3 and 13), 5 (LGs 5 and 15), and 9 (LGs 9 and 19), in descending order of frequency (Supplementary Figs. 3b, 7, a and b). Overall, phase 1 exhibited substantially fewer interspersed genotyping patterns than phase 2. This difference may be expected given the unequal marker density between phases and the inheritance of chromosomes from the wild-species-derived neoallotetraploid parent in phase 2. Notably, the LGs containing the highest numbers of individuals with interspersed genotyping patterns also showed the most extreme “doubled” LG behavior.

With this in mind, we followed the intuition that these chromosome-scale interspersed genotyping patterns represented some chromosomal entity, or biological circumstance, that did not follow standard assumptions, and that this might underlie the doubled LG patterns. To test this, 18 BC_1_ progenies with pronounced interspersed genotyping on LG14 in the phase 2 map were excluded, and linkage mapping was repeated. Satisfyingly, this eliminated the doubled LG patterns (Fig. 4b and Supplementary Fig. 3a).

Then, to investigate the nature of the entity or circumstance underlying the interspersed genotyping patterns, we performed whole-genome sequencing on BC_1_ progenies with (Ind 47 and 62) and without (Ind 33 and 39) the interspersed genotyping patterns in LG14. In addition, BC_1_F_2_ progenies Ind 40.3 and 40.4, descendants of BC_1_ individual Ind 40, which also exhibited interspersed genotyping on LG14, were sequenced.

Sequencing revealed that individuals with interspersed genotyping patterns had unbalanced genomic compositions on the same chromosomes. Specifically, large-scale AAAB configurations were detected in chromosome set 4 in Ind 47 and 62 that displayed interspersed genotyping, but not in Ind 33 and 39 that had normal genotyping patterns (Fig. 5, a–d). Similarly, BC_1_F_2_ progenies Ind 40.3 and 40.4 showed the same large unbalanced AAAB configuration in chromosome set 4 (Fig. 5, e and f).

**Fig. 5.**
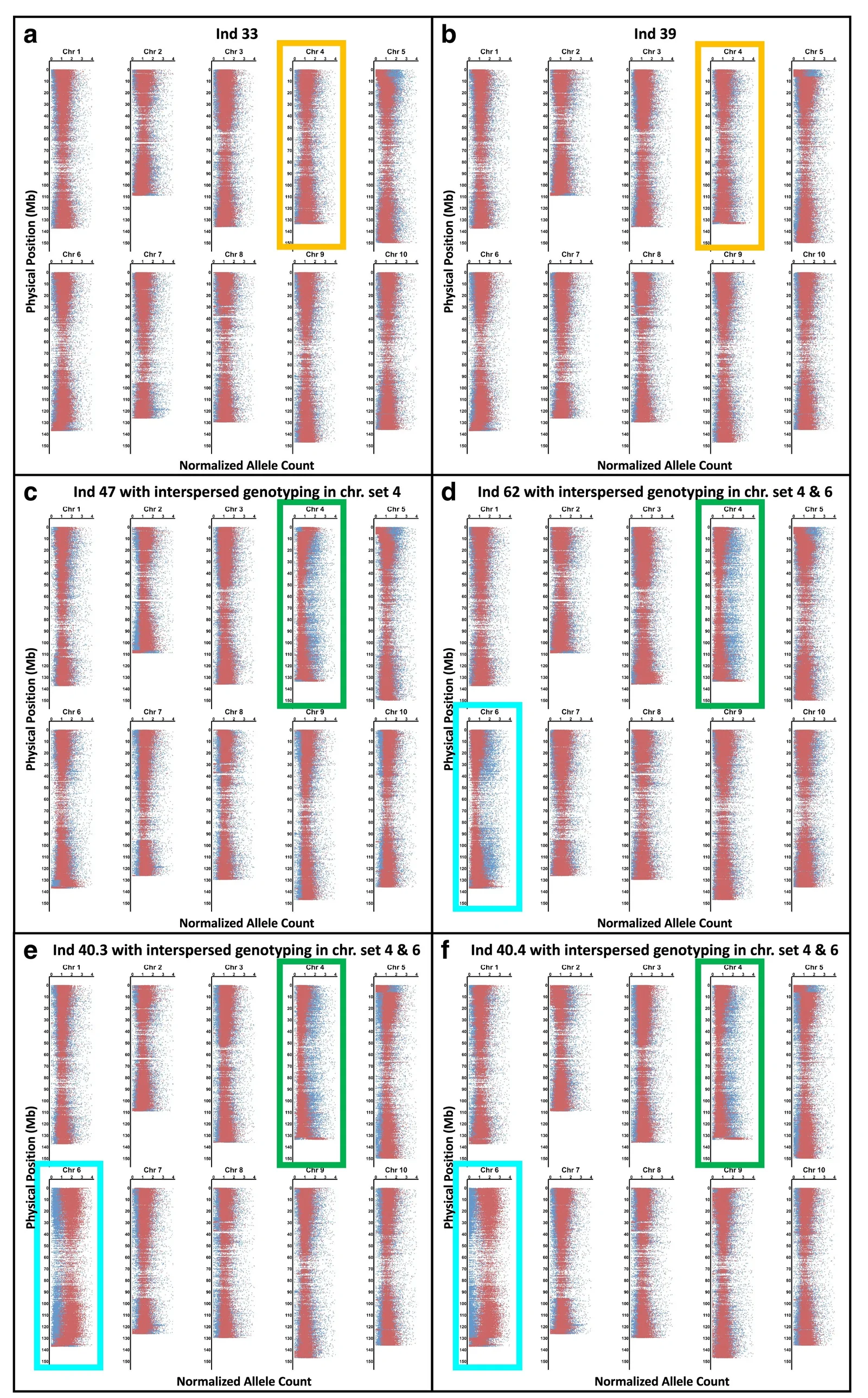
Read density plots identifying unbalanced genomic compositions. Density plots illustrating read counts by chromosome for sequenced genotypes distinguishing between *Arachis duranensis*-like (AA-genome; blue) and *A. ipaënsis*-like (BB-genome; red) subgenomes. Chromosomes with a balanced composition of A and B subgenomes (AABB genome) show read counts of 1 for both A and B reads. In contrast, chromosomes with genome shifts toward either *A. duranensis* or *A. ipaënsis* display varying values, with common scenarios being A = 1.5 and B = 0.5 for the AAAB-genome, and A = 0.5 and B = 1.5 for the ABBB-genome. a) and b) show Ind 33 and Ind 39, both with a regular (balanced) chromosome set 4, highlighted in yellow. c) Shows Ind 47, which displays an unbalanced chromosome set 4, highlighted in green. d) Shows Ind 62, which displays unbalanced chromosome sets 4 and 6, highlighted in green and cyan, respectively. e) and f) Show the BC_1_F_2_ progenies Ind 40.3 and Ind 40.4, both derived from Ind 40, each with unbalanced chromosome sets 4 and 6, highlighted in green and cyan, respectively. These highlighted regions are consistent with the genotyping patterns detected in each individual.

Across all individuals, chromosome sets 2 to 7 exhibited individual-specific genomic composition variation, whereas sets 1, 8, 9, and 10 were largely balanced. Notably, individuals with an AAAB composition at the top of chromosome set 5 had yellow flowers (Supplementary Fig. 8). This agrees with the previously reported genetic locus for flower color (Bertioli et al. 2019) and with the dominance of the yellow wild allele carried on the A subgenome in this population.

### DR estimation in segmental allotetraploid peanut

Pericentromeric boundaries for each LG were visually estimated to allow clearer interpretation of DR event distribution (Fig. 4, a and b, and Supplementary Table 3). In both phase 1 and phase 2, DR scores were predominantly detected near the telomeric regions, consistent with previous studies (Supplementary Table 4) (Bourke et al. 2015). Specifically, in phase 1, DR events occurred at the top of LG7 (0.34 to 6.54 Mb, Ind 21), the top of LG12 (1.49 to 1.77 Mb, Ind 38), the bottom of LG15 (157.87 to 159.92 Mb, Ind 59), and the top of LG19 (0.18 to 0.43 Mb, Ind 59), with all physical positions referenced to the “Tifrunner” genome (Fig. 6a). In phase 2, DR events were consistently observed at the top of LG4 (0.19 to 3.88 Mb in the “Tifrunner” genome) in six individuals (Ind 8, 11, 40, 47, 58, and 59) (Fig. 6b). Thus, DR events were most frequent on LG4 in phase 2, whereas in phase 1 they were distributed across several LGs but always confined to telomeric regions. This difference should be interpreted cautiously because phase 2 contained more markers than phase 1, which may have increased the resolution for detecting consecutive DR-type marker patterns.

**Fig. 6.**
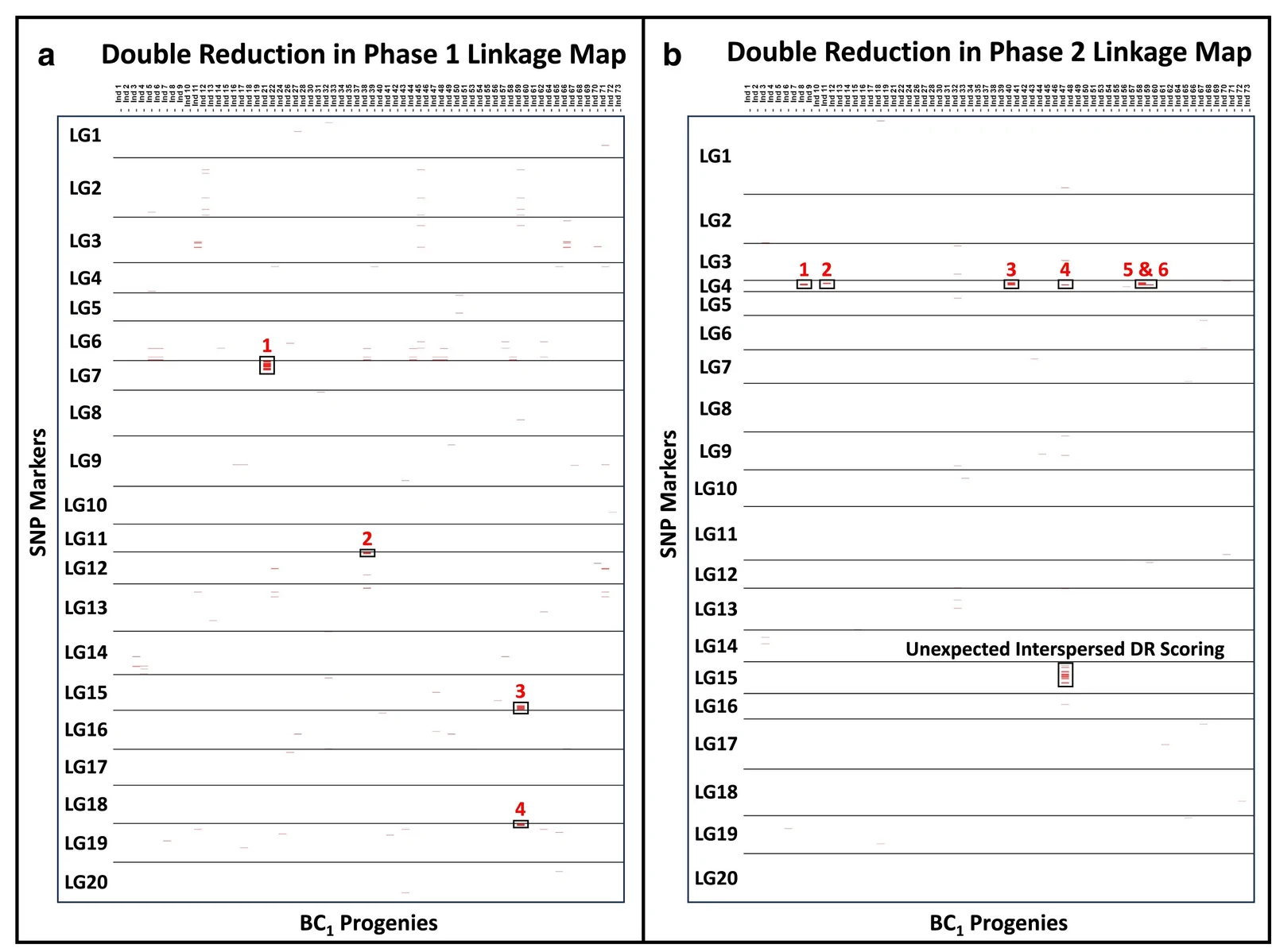
Genome-wide distribution of DR events in the BC_1_ population. In a) and b), heatmaps display DR events in BC_1_ progenies based on phase 1 and phase 2 linkage maps, respectively, with SNP markers ordered by genetic distance. Red marks indicate SNP scores consistent with DR, while regions with consecutive DR-consistent scores, identified as DR events, are highlighted with black rectangles and numbered in red. In agreement with previous reports, DR events occurred either at the top or bottom of the LGs: the top of LG7 (1), top of LG12 (2), bottom of LG15 (3), and top of LG19 (4) in the phase 1 map, and the top of LG4 (1 to 6) in the phase 2 map. In phase 2, unexpected interspersed DR scoring was observed across LG15 in Ind 47.

Among the BC_1_ progenies, 12% (8 out of 66) showed evidence of DR in either phase 1 or phase 2 linkage maps (Supplementary Table 4). Because only a subset of marker combinations was analyzed, this figure underestimates the true frequency of DR in this population. In addition, an AAAB genomic configuration was detected in LG4 of the sequenced individual Ind 47 within the DR region (3.15 to 3.88 Mb in the “Tifrunner” genome, corresponding to 4.27 to 4.97 Mb in *Arachis ipaënsis* gnm1) (Figs. 5c and  7). This result supports the theoretical expectation that DR events generate unbalanced genomic composition in progenies (Fig. 1).

**Fig. 7.**
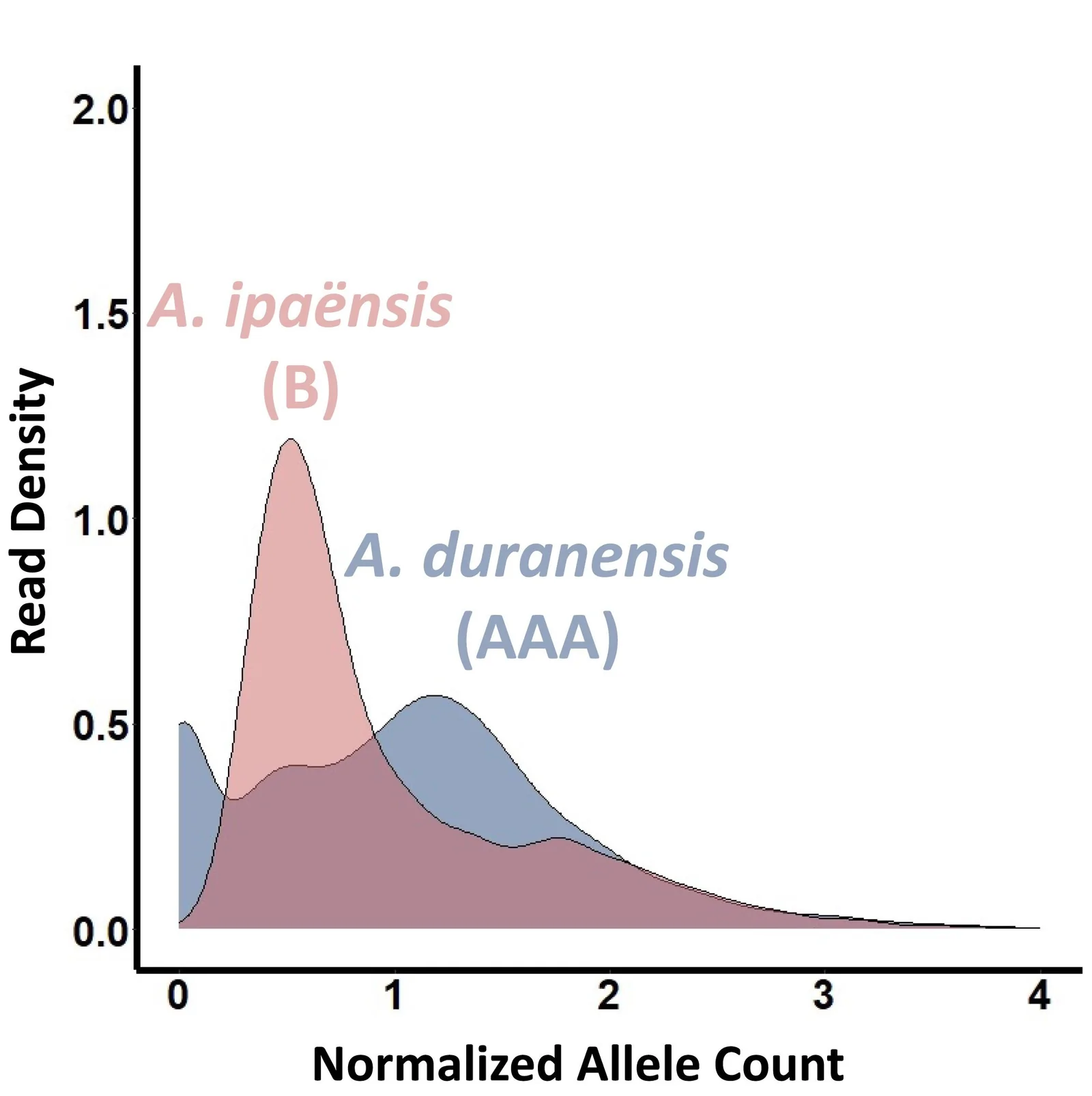
Read density evidence for DR-associated genome imbalance. Normalized allele count read density plot for the upper region of LG4 (4.27 to 4.97 Mb in *Arachis ipaënsis* gnm1; 3.15 to 3.88 Mb in the “Tifrunner” genome) of individual 47, where DR duplex scores were detected. The expected DR-derived genome composition for this region is T^a^T^a^G^a^G^b^, where “T” and “G” are arbitrary alleles, with the two T^a^ alleles provided by *A. stenosperma* (duplicated through DR), the G^a^ allele from the A subgenome of “IAC OL4,” and the G^b^ allele from the B subgenome of “IAC OL4.” The plot demonstrates a shift in genome composition toward *A. duranensis* (AAAB), as indicated by a normalized allele count of approximately 1.5 for the A subgenome and 0.5 for the B subgenome. This scenario corresponds to the genomic configuration depicted in Fig. 2, c and d.

However, Ind 47 also displayed an unexpected interspersed pattern of DR scores across LG15, including substantial presence in the pericentromeric region, an area typically not associated with DR (Bourke et al. 2015). Notably, Ind 47 did not exhibit the anticipated unbalanced genomic configuration in this region (Fig. 5c), suggesting that a different genetic mechanism, rather than DR, may underlie these patterns.

### Hidden Markov model revealed homoeologous exchange but could not confirm DR

The hidden Markov model using a tetrasomic model allowed for both bivalent and multivalent pairing. The model was run at the default error prior setting (Supplementary Figs. 9 and 10). With this setting, two individuals were predicted to have arisen from multivalent pairing in the parental meioses, namely Ind 47 on chromosome 05 and Ind 21 on chromosome 07 (Supplementary Fig. 11). However, when the process was repeated over a range of possible error priors, the maximum-likelihood results no longer predicted multivalent pairing for these (or any other) individuals (Supplementary Fig. 11). Therefore, the hidden Markov model analysis did not confirm DR. Nevertheless, it identified six individuals with patterns consistent with homoeologous exchanges on chromosomes 2, 3, 5, and 7 (Supplementary Fig. 12).

## Discussion

This study provides evidence of individual DR events in single progeny of an established allopolyploid, supporting the conclusion that DR can generate local unbalanced genomic composition (Figs. 1 and 7). DR signatures were detected in 12% of the analyzed BC_1_ peanut progenies. This indicates that DR can occur at appreciable levels in this genetic background. The events were more frequent in chromosomes inherited from the wild-species-derived neoallotetraploid parent (phase 2) than in those from the cultivated parent (phase 1). This 12% should not, however, be read as the expected frequency of DR in modern cultivated peanut. Two considerations affect the estimate, and they push it in opposite directions.

The first set of considerations suggests that the true rate in cultivated peanut may be lower than 12%. The wild-derived MagSten chromosomes segregating in the F_1_ have not been selected for disomic pairing in the way established cultivated peanut has. Their meiosis is therefore more prone to the homoeologous pairing and multivalent formation that give rise to DR. The higher DR frequency we observed in phase 2 is consistent with this, and with the greater homoeologous exchange previously reported in wild-derived peanut material (Leal-Bertioli et al. 2015a; Lamon et al. 2025a, 2025b). One caveat applies here: phase 2 also carried more markers than phase 1, so we cannot fully separate this biological effect from the greater detection resolution that more markers provide. A further source of possible overestimation is that certain alternative mechanisms can mimic DR—specifically, tetrasomic inheritance in the female parent (phase 1) or two consecutive rounds of tetrasomic inheritance in the male parent (phase 2).

The second set of considerations works in the opposite direction and suggests we may have underestimated DR. Detection was limited to a subset of the events that could in principle occur. The number of informative marker combinations was limited. Stringent dosage filtering was required to score segregation accurately. And the structure of the BC_1_ population itself allows only certain DR events to be observed at all.

Taken together, these factors leave the precise rate uncertain, but they do not undermine the individual events themselves. DR remains the most parsimonious explanation for the patterns we report, and our conservative scoring approach—which required multiple consecutive markers with consistent dosage—minimizes the risk of false positives.

All identified DR events in our study were localized to telomeric regions, in agreement with established theory and previous empirical observations (Mather 1936; Fisher 1947; Haynes and Douches 1993; Butruille and Boiteux 2000; Stift et al. 2008; Nemorin et al. 2012; Bourke et al. 2015; Ahmed et al. 2020). This localization is expected because DR requires a crossover between the focal locus and its centromere, and the probability of such a crossover increases with map distance from the centromere; it is further favored by the higher crossover frequency and multivalent formation near telomeres relative to the structurally constrained pericentromeric regions. Interestingly, we observed an unusual interspersed pattern of DR-like scores across the pericentromeric region of LG15 in Ind 47, which was not associated with an unbalanced genomic configuration in the corresponding chromosome set. Ind 47 was also initially predicted to have arisen from DR using the hidden Markov model, although this prediction was subsequently lost at a higher error prior. However, the autotetraploid hidden Markov model we employed is reliant on a high-quality integrated linkage map. Our pseudo-integrated map was visibly discordant on certain LGs including chromosome 05 (Supplementary Fig. 5), which may have affected the quality of the results. These anomalous patterns in Ind 47 may reflect a genetic mechanism other than DR. However, the limitations of the pseudo-integrated map, together with our inability to determine whether the anomaly was inherited because of the lack of progeny from this individual, preclude a firm conclusion.

A central aim of this study was to test the hypothesis, proposed in Fig. 1, that DR can generate unbalanced genomic compositions in segmental allotetraploid peanut. In Ind 47, a DR-type segregation signature—a duplex allelic score arising from a single-dosage locus—was detected in the phase 2 map across a discrete interval at the top of LG4. Sequencing of the same individual confirmed a local AAAB configuration over this region (4.27 to 4.97 Mb in *A. ipaënsis* gnm1; 3.15 to 3.88 Mb in Tifrunner), matching the theoretical prediction that a DR-derived gamete, fertilized by a standard disomic gamete, yields a locally unbalanced subgenome dosage (Figs. 1, 2, and 7). Notably, this interval lies at the leading edge of the much larger AAAB region (∼5 to 130 Mb in *A. ipaënsis* gnm1) attributable to homoeologous exchange in the same individual. We interpret the two as mechanistically distinct despite their physical adjacency: the duplex DR-diagnostic score is confined to this discrete top interval and is not present across the remainder of the large tract, indicating that the two AAAB regions arose through different processes rather than as a single continuous event. This distinction is also one of the scale—the DR-associated imbalance spanning less than 1 Mb against tens of Mb for the exchange-associated region. DR and homoeologous exchange may therefore both perturb subgenome composition in peanut, but through different mechanisms and at markedly different scales.

The biological and breeding relevance of DR in peanut is likely context-dependent. In autopolyploids, where DR is most commonly described, DR can increase local homozygosity by causing two identical-by-descent chromosomal segments to be inherited together (Butruille and Boiteux 2000; Bourke et al. 2015; Huang et al. 2019). This can affect phenotype positively or negatively, depending on the alleles and genomic region involved. In segmental allotetraploid peanut, DR may have the same effect on local homozygosity, but it can additionally alter local subgenome dosage, producing the unbalanced genomic compositions demonstrated above (Figs. 1 and 7). Such unbalanced regions may affect chromosome pairing in subsequent generations, potentially increasing the likelihood of segregation distortion, homoeologous exchange, or additional dosage imbalance.

The breeding relevance of DR is also expected to differ between wild-derived and cultivated peanut material. In wild-derived neoallotetraploid material, DR could expose or fix useful variation introduced from wild species (Lamon et al. 2025a, 2025b). In cultivated peanut germplasm, similar effects may occur, but their impact would likely be more limited because cultivated genomes are more genetically similar and generally more stabilized. Thus, DR may represent a source of genetic variation relevant for cultivar improvement, most likely in wild-derived material.

Detecting these events depended on a BC_1_ population which, despite the manual crossing that peanut's self-pollinating habit demands, was essential for generating the single-dosage-to-duplex segregation patterns DR detection requires (Moretzsohn et al. 2023). This population, together with SNP chip technology and the “fitPoly” and “polymapR” packages in RStudio, also allowed us to construct a phased linkage map for peanut, achieving high marker density compared to previous studies (Zhou et al. 2014; Leal-Bertioli et al. 2015b; Huang et al. 2016; Li et al. 2017; Han et al. 2018; Ballén-Taborda et al. 2019; Liu et al. 2020; Blas et al. 2021; Sun et al. 2022; Miao et al. 2023; Tsai et al. 2024).

Map construction and sequencing analyses also revealed that regions with frequent interspersed genotyping patterns, especially in LG14, coincided with unbalanced genomic compositions and double-LG artifacts in Marey plots. In some cases, these mapping artifacts were so extensive that they split a single LG into two distinct segments. Although we have seen similar issues elsewhere, they have not been addressed in the literature—data presumably being culled in a practice Sydney Brenner termed “Ockham's broom” (Leal-Bertioli et al. 2015a). Unbalanced chromosomal configurations likely influence linkage maps and inheritance patterns in breeding populations to a surprising degree (Lamon et al. 2025b). This study demonstrated that the identification and filtering of interspersed genotyping prior to map building reduced mapping artifacts and improved the accuracy of both map construction and downstream analyses. Together, these findings identify DR and homoeologous exchange as recurrent, mechanistically distinct sources of genomic instability in segmental allotetraploid peanut—the former introducing localized, allele-specific dosage changes and imbalances, the latter generating imbalance across large chromosomal segments. Both reshape subgenome composition and, in doing so, contribute to the genetic instability and the generation of novel allelic combinations that continue to shape this and, likely, other allopolyploid genomes.

## Data Availability

Whole-genome sequencing data can be accessed through NCBI under BioProject accession PRJNA1240332. Sequencing analysis scripts are available at https://github.com/brianabernathy/ABGD. Supplementary Figures and Supplementary Tables are available at FigShare, G3 (Bethesda) online (https://doi.org/10.25387/g3.33399301).
